# Effect of the Construction of Carbon Fiber Plate Insert to Midsole on Running Performance

**DOI:** 10.3390/ma14185156

**Published:** 2021-09-08

**Authors:** Fengqin Fu, Ievgen Levadnyi, Jiayu Wang, Zhihao Xie, Gusztáv Fekete, Yuhui Cai, Yaodong Gu

**Affiliations:** 1 Faculty of Sports Science, Ningbo University, Ningbo 315000, China; fufengqinnb@aliyun.com (F.F.); wjy970507@gmail.com (J.W.); 2 Doctoral School on Safety and Security Sciences, Óbuda University, 1011-1239 Budapest, Hungary; 3 Xtep Sports Science & Engineering Laboratory, Xtep Co. Ltd., Xiamen 361000, China; evgenabaqus@gmail.com (I.L.); zhihao.xie@xtep.com.cn (Z.X.); yuhui.cai@xtep.com.cn (Y.C.); 4 Savaria Institute of Technology, Eötvös Loránd University, 9700 Szombathely, Hungary; fg@inf.elte.hu

**Keywords:** longitudinal midsole bending stiffness, 3D kinematics, ground reaction force (GRF), footwear, carbon running shoes, performance, finite element simulation

## Abstract

In this paper, to investigate the independent effect of the construction of the forefoot carbon-fiber plate inserted to the midsole on running biomechanics and finite element simulation, fifteen male marathon runners were arranged to run across a runway with embedded force plates at two specific running speeds (fast-speed: 4.81 ± 0.32 m/s, slow-speed: 3.97 ± 0.19 m/s) with two different experimental shoes (a segmented forefoot plate construction (SFC), and a full forefoot plate construction (FFC)), simulating the different pressure distributions, energy return, and stiffness during bending in the forefoot region between the SFC and FFC inserted to midsole. Kinetics and joint mechanics were analyzed. The results showed that the footwear with SFC significantly increased the peak metatarsophalangeal joint (MTPJ) plantarflexion velocity and positive work at the knee joint compared to the footwear with FFC. The results about finite element simulation showed a reduced maximum pressure on the midsole; meanwhile, not significantly affected was the longitudinal bending stiffness and energy return with the SFC compared to the FFC. The results can be used for the design of marathon running shoes, because changing the full carbon fiber plate to segment carbon fiber plate induced some biomechanical transformation but did not significantly affect the running performance, what is more, reducing the peak pressure of the carbon plate to the midsole by cutting the forefoot area of the carbon fiber plate could be beneficial from a long-distance running perspective for manufacturers.

## 1. Introduction

It was notable that track shoes such as the Nike Vaporfly 4% (VF) shoe combine both advances in midsole thickness and longitudinal bending stiffness (LBS) to reduce energy loss by about 4% for runners [1,2,3,4], which contributes to an improved running performance [4]. Runners wearing the VF shoe broke world records in the full-marathon, half-marathon, and 100 km distances, and so on [5]. It was not yet understood whether the midsole material [3,6], midsole construction [5], or shape of carbon-fiber plate [7,8] contribute more to these ‘racing running shoes’. The full-length embedded carbon fiber plate to midsole would increase the LBS of the shoe [9,10], reducing running economy by about 1% [6].

From biomechanical perspectives, several kinds of research indicated that increasing the LBS of running footwear may significantly reduce energy loss at the metatarsophalangeal joint (MTPJ) [11,12,13,14]. The energy loss might be caused by the MTPJ changes on account of an increased LBS [12,15,16,17], and increased peak plantarflexion moment [12,15]. There was no consistent conclusion about it. Healey and Hoogkamer [18] highlighted that there was no significant effect on the energy savings in the Nike Vaporfly 4% by decreasing the LBS which indicated that the function of the plate in the 4% energy savings is the limitation. In addition, there was a new effect on running mechanics that the influence of the curved carbon fiber plate inserted into the midsole worked as a ‘teeter-totter’ [8]. The plate stiffens the MTPJ and works as a lever to decrease the work rate at the ankle [19]. The principle was that the point of application of the ground reaction force moves anteriorly and towards the front end of the curved carbon fiber plate during the second half of ground contact [8].

It was still worth mentioning that Burns and Tam [5] introduced the midsole thickness as the main footwear characteristic that has advantages to improve running performance. Increasing the midsole thickness could protonate the effective leg length of the runner such as the VF shoe which has a 31 mm heel height [20]. It could decrease energy loss for the runner by increasing an 8 mm effective leg length [21,22]. Besides, some researchers also figured out that the effect of midsole thickness is about 1% for running economy [23]. The VF made runners trend more to midfoot or forefoot strike and has high requirements for the runner’s muscle strength because of its high rearfoot thickness and the strong propulsion structure of forefoot [24]. The previous research on the foot strike patterns demonstrated that the rearfoot strike pattern is mainly used among the prolonged runners in road races, with percentages ranging from 74.9% of runners in a professional half-marathon race [25], to over 90% of amateur runners in marathon distance events [26,27]. The Xtep innovation R&D center thus created a pair of racing shoes that reduced the thickness of the midsole but retained the curved carbon fiber plate to meet the needs of marathon runners of different levels. According to the pilot work from the Xtep lab, reducing the thickness of the midsole induced that marathon runners felt too hard on the forefoot area if they continued to run after 30 km when wearing running shoes with a full carbon fiber plate.

In summary, it was valuable to do further research about the construction of the forefoot plate such as adjusting the full forefoot plate construction (FFC) to segmented forefoot plate construction (SFC). The effects of the forefoot construction of the carbon fiber plate have not been investigated, and it can likely be very hard to investigate the pressure distribution on the plate or midsole through human trials [28]. In recent years, finite element (FE) methods are commonly applied in biomechanical research of the lower extremity due to their ability to process the complex geometry structures for both static and dynamic analysis [29,30,31].

This study aimed to research the effect of the construction of the forefoot plate combined with the running biomechanics and finite element (FE) simulation. Based on previous literature, it was hypothesized that (1) the SFC model has a lower LBS compared to the FFC model which increases the angle of MTPJ dorsiflexion and would potentially increase the amount of energy loss at the MTPJ; (2) the SFC model has a lower maximum pressure on the forefoot area compared to the SFC model.

## 2. Materials and Methods

### 2.1. Participants

Fifteen male runners (mean (SD) age: 34.93 (10.25) years, height: 1.70 (0.05) m, weight: 61.47 (45.59) kg, BMI: 21.22 (1.77) kg/m2) joined in this research. All of the participants were recruited from the Xiamen running club and identified themselves as rearfoot strike runners. Participants were free from injury for at least six months before this study. All participants had been confirmed in foot size (EU 41 ± 0.5) by the Brannock Device (The Brannock Device Co., Syracuse, NY, USA) before the official test.

### 2.2. Experimental Footwear

**Figure 1:**
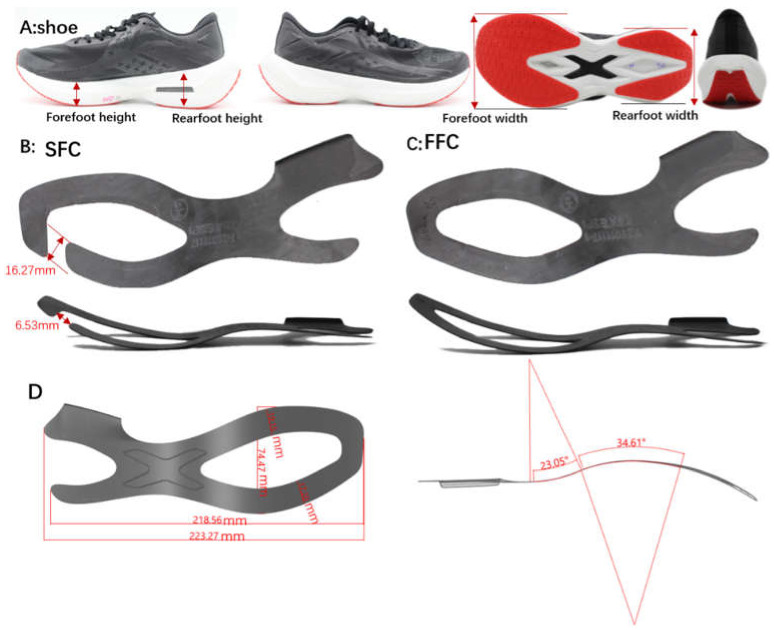
(**A**): Experiment shoe (Forefoot height: vertical thickness at 12% of external length, Rearfoot height: vertical thickness at 75% of external length, offset: offset = rearfoot height − forefoot height); (**B**): the forefoot area of carbon fiber plate (carbon fiber plate was made up of 63 % carbon fiber and 37% epoxy resin fiber) was designed to a segment construction inserted to midsole (SFC), (**C**): the forefoot area of carbon fiber plate was designed to a full construction inserted to midsole (FFC)); (**D**): the information about the geometry and dimensions of the carbon plates.

There were two kinds of experimental footwear used in this research: (rearfoot height: 26 mm, forefoot height: 18 mm, offset: 8 mm, rearfoot width: 76.5 mm, forefoot width: 102 mm, midsole material: foam in hardness 50 asker C, outsole material: rubber in hardness: 62 asker A, differing in their construction of carbon fiber plate (SFC: 1 mm thick carbon fiber plate with segmented forefoot plate construction, FFC: 1 mm thick carbon fiber plate with full forefoot plate construction) inserted in the midsole (Figure 1). Mechanical flexion measurements fixed the forefoot area in the location of 70% foot length (heel to toe), then bending with 45 degrees was performed by applying a dynamic shoe flexor device (Brentwood, NH, USA) to measure the shoe LBS and energy return [32], last, measuring the force on the forefoot area by a pressure sensor.

### 2.3. Methodology

#### 2.3.1. Finite Element Simulation

**Figure 2:**
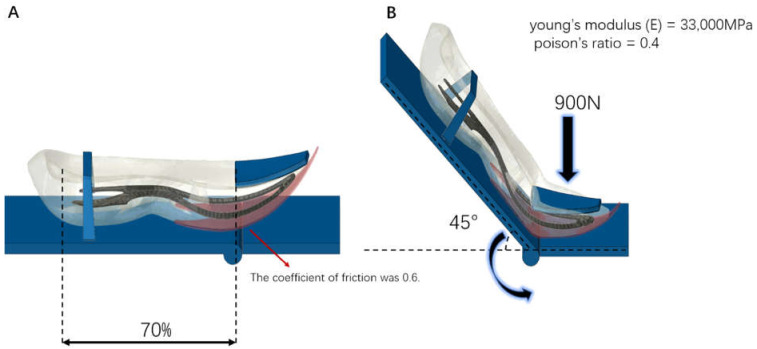
Showed the finite element simulation (**A**): The initial position, (**B**): Conditions imposed by finite element simulation).

In this study, the outsole, midsole, and two kinds of carbon fiber plate have been modeled in 3D based on an industrial 2D shoe design drawing with Rhino 6 CAD software (Robert McNeel & Assoc, Seattle, WA, USA). A meshing of the shoe has been done with ABAQUS software by this CAD model (Dassault Systemes Simulia Corp, Johnston, RI, USA) that the discretization was 2.7 mm. All of the solid parts were assembled into a whole sole model, then imported into the FE package ABAQUS (Dassault Systemes Simulia Corp, Johnston, RI, USA) to develop the numerical model. To simulate the flexion mechanical test, the sole model was firstly positioned on two rigid plates, which correspond to the virtual flexing machine: fixed and flexing one. The sole was camped to the fixed plate by applying a 900 N to toe clamp at 70% foot length (heel to toe) while the heel end is on the flexion plate (Figure 2) and the angle of flex was 45 degrees. The coefficient of friction between the sole and plates was 0.6. In this study, the midsole was made of Polyetherblockamide foam (Pebax^®^, UBESTA, Yubu Xingchan Co., Ltd.,Yubu, Japan); thus, to determine mechanical properties for finite element analysis, this material was tested at quasi-static rates by using a universal material test machine. Compression tests were performed according to the ASTM-575 standard by using cylinder specimens (diameter: 28.6 mm; thickness: 12.5 mm) at a speed of 10 mm/min. The specimen density was 0.12 g/cm^3^. Force–displacement data were obtained from the quasi-static tests and converted to stress-strain data by using the sample dimensions. The Ogden hyper foam material model was chosen to represent the non-linear response of the Pebax^®^ foam obtained from the experiments. This model describes a compressible and nonlinearly elastic behavior and its strain energy density function *U* in terms of generalized strain was:

(1) $$U=\sum_{i=1}^{N}\frac{2u_{i}}{\alpha_{i}^{2}}[\lambda_{1}^{\alpha^{i}}+\lambda_{2}^{\alpha^{i}}+\lambda_{3}^{\alpha^{i}}-3+\frac{1}{\beta_{i}}\left(J_{e1}^{-\alpha_{i}\beta_{i}}-1\right)]$$

The hyper foam material constants for Pebax^®^ foam were *μ* = 0.28, *α* = 5.177, Poisson’s ratio = 0.125. To determine the mechanical properties of the reinforced carbon fiber plate, three-point bending test was carried out using the material testing machine with a speed of 1 mm/min. The test samples were prepared according to ASTM-D790: 1 mm thickness, 18 mm width, and 80 mm length strips were cut from an original plate with the help of an electrical power saw. The specimen density was 1.1 g/cm^3^. From the mechanical test, Young’s modulus (E), was obtained = 33,000 MPa and Poisson’s ratio = 0.4. The sole, made of foam, was discretized using tetrahedral elements with an average size equal to 2.7 mm. The carbon fiber plate was discretized also, using tetrahedral elements with an average size equal to 1 mm. A convergence study has been performed to confirm if the mesh density is acceptable. Finally, the simulation was performed in Abaqus using the Dynamic Explicit solver. Peak torque (Nm), stiffness (Nm/deg) and energy return (%), contact pressure on the plates (MPa) were calculated.

#### 2.3.2. Biomechanical Data Collection

Participants performed eight valid right foot rearfoot strike running trials per testing shoe on a 145 m concrete indoor running loop. We hoped that this pair of shoes could be suitable for different types of runners, not only for professionals but also for amateur runners, so we chose a fast and slow speed for all tests. A valid trial was one within the specified velocity range (fast speed: 4.81 ± 0.32 m/s, slow speed: 3.97 ± 0.19 m/s) and made up of the whole right foot contacting the force plate area. Before, data collection participants warmed up for about five minutes and were acquainted with the target speed and shoe conditions by running 2 laps in each shoe condition. Upon failing to match the required speed in the first two laps, further familiarization laps were performed as necessary. For GRF and 3D kinematic measurements, participants ran across a set up of three consecutive and flush into the floor force plates (combined dimensions 270 × 60 cm, 1000 Hz (AMTI, Watertown, MA, USA)) in each shoe condition. The test sequence of shoes was randomized for each participant. The two-timing gates 8 m far from the middle force plate were used to record the running speed (Smart speed, Burbank, CA, USA) set 8 m apart, centering the middle force plate. Right leg kinematics were collected at 250 Hz and were collected using a 10-camera motion analysis system in a capture volume of 4.0 × 1.0 × 1.5 m (Vantage 5, Vicon, Metrics Ltd., Oxford, UK). The marker set was according to the calibrated anatomical systems technique [33]. The right thigh, the right shank, the right foot (forefoot and rearfoot) were defined as segments by attaching retro-reflective markers of fourteen millimeters in diameter on the skin of the right and left anterior superior iliac spine (ASIS), the right and the left posterior superior iliac spine (PSIS), the right greater trochanter, the medial and lateral epicondyle of the femur, the medial and lateral malleolus, as well as attached to the shoe, representing the first and fifth metatarsal heads and second toe. Four marker tracking clusters were attached to the lateral side of the thigh and the lateral side of the lower leg [34]. The extra reflective markers were added to the distal, proximal heel, and lateral rearfoot, respectively, and were defined as shoe-mounted tracking markers [35]. A static trial was conducted before data collection; all study procedures about biomechanical data collection were similar to the published paper [36], both of them performed in the Xtep science lab. In the trial, valid data could be used when the first impact peak and shoe ground angle more than zero appeared. We used the Vicon Nexus 2.7 and Visual3D (C-Motion, Germantown, MD, USA) to process the collected experimental data. A fourth-order low pass Butterworth filter was used with a cut-off frequency of 100 Hz (kinetic) and 10 Hz (kinematic) [37]. The XYZ Cardan sequence was used to calculate lower limbs’ kinematic and kinetic data, in which X represents flexion–extension, Y represents abduction–adduction, and Z represents internal–external rotation [38]. The angle, the angular velocity, the ground reaction force and the work of the hip, the knee, the ankle and the MTP joints of the right lower limb were measured during the stance phase using Visual3D (C-Motion, Germantown, MD, USA).

### 2.4. Statistical Analysis

Statistics were processed by SPSS (24, IBM Corp., Armonk, NY, USA). Shapiro–Wilk tests were adopted in this study. A 2 × 2 (factors: running speed, running shoes) within-subjects factorial repeated-measures analysis of variance (RM ANOVA) was selected to evaluate the main effects and the interaction of these factors on the biomechanical variables. Statistical Alpha levels were set to 0.05. The alpha levels were adjusted to < 0.003 according to post-hoc pairwise comparisons with a Bonferroni correction when variables showed a significant main or interaction effect. Partial eta squared estimates (ηp^2^) were calculated for statistically significant variables.

A Statistical Parametric Mapping (SPM) technique [39,40] assessed the main effects of ‘running shoes’ and ‘running speed ‘factors and their interaction, and SPM tests were calculated with the SPM1D v0.4 for MATLAB (www.spm1d.org (accessed on 1 March 2021), [39]). The significance level was set at 0.05 for all statistical tests.

## 3. Results

**Figure 3:**
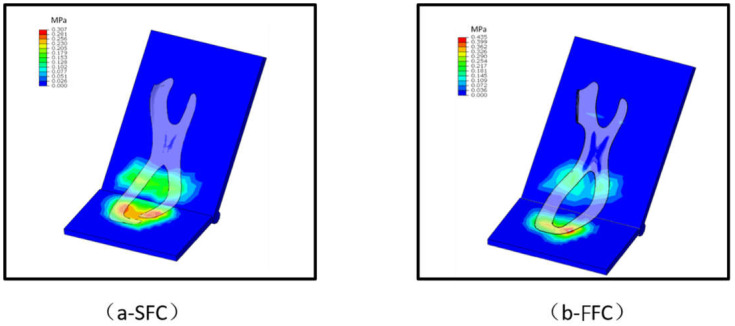
The pressure on the SFC and FFC during bending.

The FE simulation showed that the maximum pressure on the forefoot of SFC (0.307 MP) was lower than that on the FFC (0.435 MPa) (Figure 3), but the results from the forefoot flexion scores and bending simulation indicated that there were no effects between SFC and FFC in LBS and energy return (Table 1).

**Figure 4:**
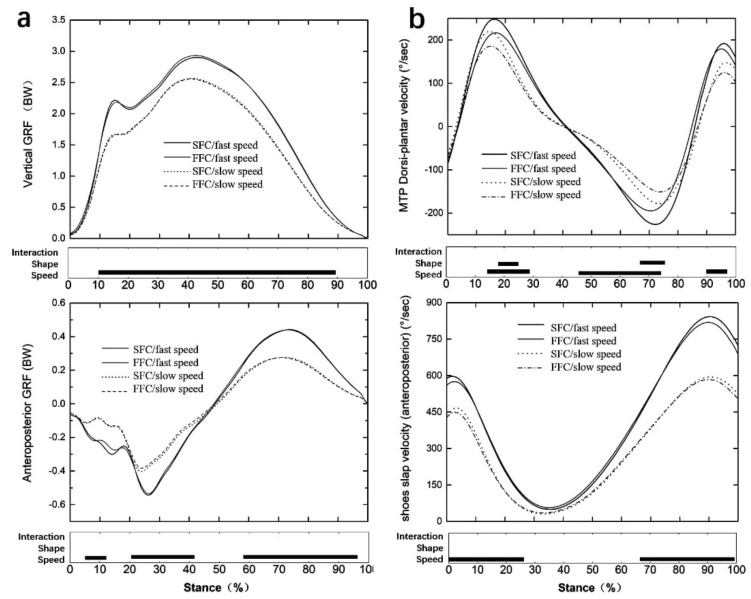
Vertical ground reaction force time (**a**) and anteroposterior ground reaction force time (**b**) (weight−normalized) (shoe slap velocity showed the shoe ground velocity).

**Figure 5:**
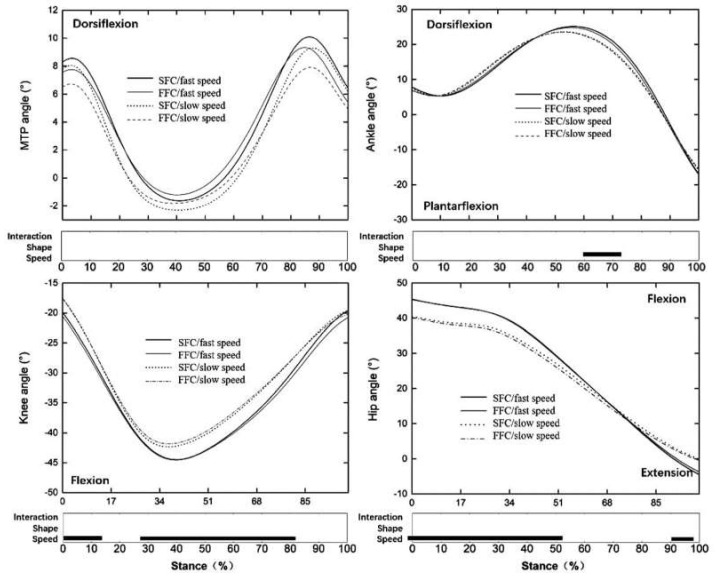
Lower limb joint angles time−normalized.

**Figure 6:**
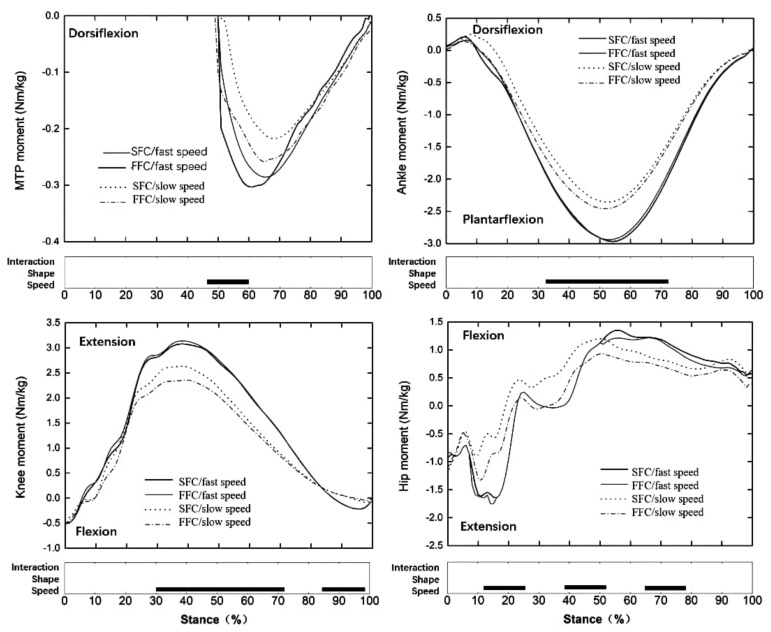
Lower limb joint moment time and weight−normalized. Note: The significant main effects of the interaction, the location, and the speed are highlighted (black horizontal bars at the bottom of the figure) during the stance phase of running.

**Figure 7:**
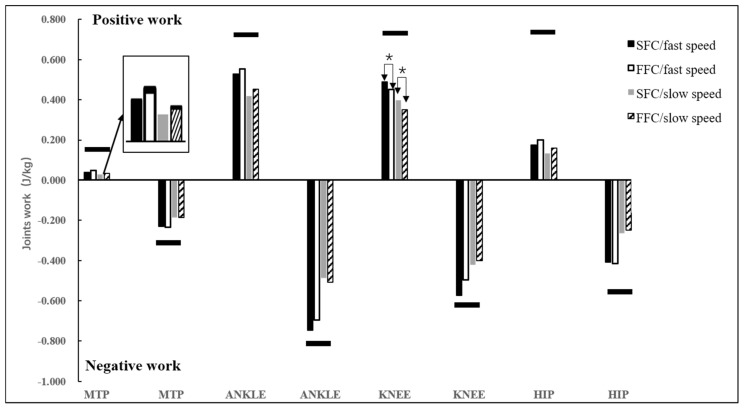
Joints work and showed significant main effects of the interaction; the black horizontal bars showed significant main effects of speed; and the * showed significant main effects of the construction of the carbon fiber plate.

The result showed that the vertical and anteroposterior GRF (Figure 4a), ankle, knee, and hip range of motion (Figure 5), the moment at each lower limb joint (Figure 6), MTPJ, and shoes slap velocity (Figure 4b), positive and negative work at each lower limb joint (Figure 7) of faster speed (4.81± 0.32 m/s), were bigger than with the slower speed (3.97 ± 0.19 m/s) in both experimental shoes (*p* < 0.05).

As for the effect of the construction of the carbon fiber plate, the SFC induced more MTPJ Dorsi-plantar velocity from 18% to 26% (*p* < 0.05) and 67% to 78% of the stance phase (*p* < 0.05) compared to the FFC (Figure 4b). The positive joint work at the knee joint (*p* = 0.038, ηp^2^ = 0.178) was larger for the SFC compared to the FFC (Figure 7). There were no significant differences between SFC and FFC at ground contact time, breaking phase time, and some variables for MTPJ, such as MTPJ negative work, MTPJ Dorsi-plantar range of motion, and so on (Table 2). In addition, there is no effect from the interaction between the construction of the carbon fiber plate and the running speed.

## 4. Discussion

This research aimed to investigate the effect of the construction of a carbon fiber plate. In contrast to our first hypothesis, differences in the construction of the carbon fiber plate did not induce differences in shoe LBS during bending (Table 1 and Table 2). This is inconsistent with the previous result which has shown that cutting the carbon fiber plate would reduce the shoe LBS due to the mechanical behavior-changing of the shoe midsole [18]. What’s more, the results of mechanical and finite element analysis showed that changing the construction of the carbon fiber plate on the forefoot area could also not affect the energy return. This could be due to the adjustment in the carbon fiber plate being too small to cause a noticeable difference. These were some differences between experimental data and simulation results, even though the model could display realistic trends in general, it overestimated the energy return of the midsole measured in the flexion machine, and the reasons causing the overestimation are discussed below. In the analysis, the material models applied were a simplistic representation of the complex behavior of each material in response to loading. For example, the modeled carbon plate did not have defined viscoelastic properties and thus no means for energy dissipation. In the analysis, the representativeness of the loading conditions used also has a degree of uncertainty. Firstly, the energy return was calculated for a single trial, and the model output was compared to average values measured across 65 trials. The accuracy of the calculated results was also dependent on the force which was applied to the toe clamp to hold the sole during flexion. During flexion test motion of the toe, the clamp was observed, while for the simulation, when flexion was applied, fixation was fixed. The interaction between the footwear parts and the footwear–flexion machine is another area that contained several significant simplifications. The friction coefficient used in the analysis, for the whole model, was taken to be equal to 0.6. While not investigated, it is hypothesized that, by using a different friction coefficient between different parts, improved results could also be achieved, as opposed to the same coefficient used in the current methodology. Finally, the exclusion of the outsole, insole, and upper, from the FE model, could have resulted in an overestimated energy return of the sole. Even the methodology applied has reported limitations; there are similarities between the results predicted with the analysis and those measured from flexion tests, with comparable trends in the peak torque of the soles observed. While not perfect, the model was still considered valuable as a comparative tool, to evaluate the peak torque, energy return, sole stiffness, stresses, and strains that might occur in future footwear designs. Nevertheless, changing the construction of the carbon fiber plate derivaized several biomechanical changes during running: For example, SFC increased peak of the MTPJ plantarflexion velocity, and the positive work at the knee compared to FFC.

From biomechanical perspectives, the MTPJ joint is a possible target area for the application of improving running performance. There was an increased peak of MTPJ plantarflexion velocity with the SFC compared to FFC (Figure 4b). The major factor was that the carbon fiber plate might work as a torsional spring, which stored and returned elastic energy as the MTPJ joint underwent rotational deformation during the ground contact in running, and cutting the carbon fiber plate would weaken this function of the torsional spring [41,42,43]. In addition, some studies have shown that carbon fiber plates have the ability to store and return elastic energy, which is presented by more positive work performed at the MTPJ [12,17,44], and there was a redistribution of positive lower limb joint work from the knee to the MTPJ when increasing the midsole bending stiffness [44]. There was more positive work at the knee joint with the SFC compared to FFC in this study. The main factor was that the midsole bending stiffness deformation of experimental shoes was not enough to lead to the work redistribution on the lower limb joint.

In line with our second hypothesis, the maximum pressure on the forefoot area of the plate was lower with the SFC compared to the FFC during the bending simulation (Figure 3); in other words, it would reduce the maximum pressure by about 29.4% on the midsole each step when adjusting the FFC to SFC. This suggests that it is of importance to take the construction of the carbon fiber plate into account when footwear manufacturers plan to design a marathon shoe, because the racing shoes embedded in the carbon fiber plate will bend probably between 30,000 and 40,000 times during a prolonged run such as a full marathon.

Two kinds of running speed (fast speed: 4.81 ± 0.32 m/s, slow speed: 3.97 ± 0.19 m/s) induced those significant changes in this study which were in line with those previously observed [45]. The results showed that the fast speed significantly increased vertical and propulsive GRF, increased ankle, knee, and hip joint range of motion, and increased moment and work in all lower limb joints, giving more MTPJ angular velocity and shoes slap velocity compared to the slow speed. There is no effect of the interaction between the construction of the carbon fiber plate and the running speed (Figure 4, Figure 5 and Figure 6).

### Limitations and Future Directions

Ideally, we have placed the foot marker on the shoe to investigate the MTP joint, however, the results concerned the shoe flexion, angular velocity, and moment, work at the location of the MTP joint. Further, we should place the marker on the skin of the foot to better research the working mechanism of the MTP joint.

In addition, our shoes were only tested on males running at two different speeds (fast speed: 4.81 ± 0.32 m/s, slow speed: 3.97 ± 0.19 m/s). Hoogkamer et al. [3] and Barnes and Kilding [16] found that metabolic savings in the VF shoes were consistent across speeds from 3.8 to 5 m/s which include our testing speed. Further, differences in gender, weight, and shoe size can differently influence the plate on running mechanics, which should be evaluated in future research. There was evidence that the manufacturer should design the footwear according to the runners who have obvious differences in functional needs and running goals [46,47,48,49,50]. Future work should aim to concern the subjective comfort of runners after long-distance running. Future research should further investigate how different characteristics of shoes change the running economics and biomechanics by systematically evaluating one shoe feature at a time.

## 5. Conclusions

The study showed that adjusting the full forefoot plate construction to segmented forefoot plate construction induced some biomechanics changes, such as more MTPJ plantarflexion angular velocity and more positive work at the knee joint but did not affect the work at the MTPJ. In addition, the results in finite element simulation provided practical evidence for footwear manufacturers that could be beneficial from a long-distance running perspective by reducing the maximum pressure on the midsole without significantly affecting the longitudinal bending stiffness.

Future studies should include endurance tests and plantar pressure experiments to provide further assessments of the effect of the construction of carbon fiber plate to meet the need of runners.

## Figures and Tables

**Table 1:** The forefoot flexion scores for experimental shoes with the same insole and the FE simulation variables for experimental carbon fiber plate.

Measurement Method	Variable	Experimental Shoe Condition	
		SFC	FFC
Weight(g)		184.17	187.92
Forefoot flexion	Peak torque (Nm)	16.50	15.44
	Stiffness (Nm/deg)	0.370	0.369
	Energy return (%)	33.97	34.66
FE simulation	Peak torque (Nm)	13.54	13.68
	Stiffness (Nm/deg)	0.301	0.304
	Energy return (%)	64.08	64.82

**Table 2:** The biomechanical variables for MTPJ and spatiotemporal parameters between SFC and FFC.

Variables	SFC(F)	SFC(S)	FFC(F)	SFC(S)	Main Effect (Construction)	Main Effect (Speed)	Interaction Effect	Effect Size (ηp^2^) F	Effect Size (ηp^2^) s
ground contact time (ms)	185.13 ± 20.13	231.42 ± 11.44	184.69 ± 22.80	227.63 ± 16.48	p = 0.636	p = 0.000	p = 0.147	0.034	0.094
breaking phase time (ms)	92.552 ± 0.01	117.88 ± 0.00	91.495 ± 0.020	113.696 ± 0.006	p = 0.964	P = 0.009	p = 0.557	0.234	0.121
proplusion phase time(ms)	92.583 ± 1.547	114.532 ± 2.30	93.196 ± 2.182	115.012 ± 2.304	p = 0.487	p = 0.004	p = 0.219	0.052	0.059
mpj plantarflexion velocity (sagittal) (°/sec)	255.33 ± 29.04	201.55 ± 32.69	212.60 ± 39.14	171.64 ± 34.26	p = 0.015	p = 0.001	p = 0.216	0.341	0.287
MTP neagitve work(J/kg)	0.052 ± 0.006	0.049 ± 0.008	0.042 ± 0.007	0.040 ± 0.002	p = 0.123	p = 0.553	p = 0.339	0.176	0.073
MTP dorsiflexion angle at toe-off (°)	7.29 ± 2.54	6.81 ± 2.16	5.76 ± 2.14	5.35 ± 2.09	p = 0.478	p = 0.248	p = 0.363	0.173	0.131
MTP range of motion(sagittal) (°)	13.46 ± 1.50	12.82 ± 2.04	12.09 ± 1.84	11.24 ± 2.27	p = 0.135	p = 0.092	p = 0.107	0.254	0.191
mpj dorsiflexion angle (sagittal) at pp (°)	7.63 ± 3.03	4.61 ± 2.69	6.81 ± 2.80	3.88 ± 2.33	p = 0.453	p = 0.002	p = 0.410	0.179	0.142
mpj dorsiflexion angle (sagittal) maximum (°)	11.81 ± 3.11	10.92 ± 2.89	10.85 ± 2.70	9.59 ± 2.13	p = 0.297	p = 0.296	p = 0.123	0.116	0.114

**Note:** pp presented the peak propulsion force.

## Data Availability

The data presented in this study are available on request from the corresponding author.
